# Molecular Dynamics Simulation of the Rejuvenation Performance of Waste Cooking Oil with High Acid Value on Aged Asphalt

**DOI:** 10.3390/molecules29122830

**Published:** 2024-06-14

**Authors:** Zhiyu Wang, Qiang Pei, Kunjie Li, Zhonghui Wang, Xiaodong Huo, Yongwei Wang, Xudong Zhang, Shaoqi Kong

**Affiliations:** 1 Department of Chemical and Materials Engineering, Lyuliang University, Lyuliang 033001, China; wangzhiyu-1982@163.com (Z.W.); wangzhonghui202405@163.com (Z.W.); huoxiaodong840701@163.com (X.H.); wgreat@126.com (Y.W.); 2 Institute of New Carbon-Based Materials and Zero-Carbon and Negative-Carbon Technology, Lyuliang University, Lyuliang 033001, China; 3 Shanxi Transportation Technology Research & Development Co., Ltd., Taiyuan 030032, China; peiqiang0615@163.com; 4 Huaxin Gas Group Co., Ltd., Taiyuan 030032, China; 5 Shanxi Institute of Emergency Management, Taiyuan 030021, China; 996631@163.com; 6 College of Mining Engineering, Taiyuan University of Technology, Taiyuan 030032, China

**Keywords:** WCO, rejuvenation effect, rejuvenation mechanism

## Abstract

Waste cooking oil’s (WCO’s) potential as a rejuvenator of aged asphalt has received attention in recent years, with the acid value of WCO affecting its rejuvenation effect. This study explored the rejuvenation effect of WCO with a high acid value on aged asphalt by using molecular dynamics simulation. First, the representative molecules of WCO with a high acid value and asphalt were determined. The rejuvenation effect of WCO on aged asphalt was analyzed by adding different contents of WCO to an aged asphalt model. The effect of WCO on the thermodynamic properties of the aged asphalt was analyzed. The results show that WCO can restore the thermodynamic properties of aged asphalt binder to a certain extent. Regarding the microstructure of rejuvenated asphalt, WCO molecules dispersed around asphaltenes weakened the latter’s aggregation and improved the colloidal structure of the aged asphalt. In terms of interface adhesion properties, WCO can improve the adhesion properties between asphalt binder and SiO_2_, but it has limited influence on water sensitivity. The results allowed us to comprehensively evaluate the rejuvenation effect of WCO with a high acid value on aged asphalt and to explore its rejuvenation mechanism.

## 1. Introduction

Due to its economic and environmental benefits, using a high content (>30%) of recycled asphalt pavement (RAP) is becoming increasingly popular in sustainable pavement construction [1,2]. However, asphalt aging has always been the main problem affecting RAP application [3], as it can cause various defects such as the cracking, fatigue, and stripping of asphalt pavement [4,5]. The use of a rejuvenator was considered to be the best way to improve performance defects and to increase the proportion of RAP [6,7]. The physical properties of waste cooking oil (WCO) are similar to the light components of asphalt, which have good compatibility with asphalt and can be used to adjust imbalances in aged asphalt components [8]. In recent years, WCO has received much attention as a rejuvenator of aged asphalt [9]. Most studies have been based on the macroscopic performance of physical, chemical, and rheological properties, and the mechanical properties of asphalt binders and asphalt mixtures to evaluate the rejuvenating effects of WCO on RAP [3,8,10,11,12,13]. The main component of cooking oil is triglycerides (TG), which undergoes various chemical processes such as hydrolysis, oxidation, polymerization, and cleavage during the cooking process, resulting in the gradual hydrolysis of TG to fatty acids (FA), with the acid value gradually increasing. Zhang [14] found that the rejuvenation effect of WCO was related to its acid value.

In essence, the molecular interaction between asphalt components and rejuvenators dominates the performance of rejuvenated asphalt [15,16]. A rejuvenator with excellent performance not only softens the aged asphalt through dilution but also, more importantly, reduces the agglomeration of aged asphaltenes through chemical action and restores the colloidal structure of the asphalt, thus realizing the real rejuvenation of the aged asphalt [17]. Therefore, studying the effects of WCO on the microstructure of aged asphalt at a molecular scale can solve the key problem of aged asphalt rejuvenation.

The advantage of molecular dynamics (MD) simulation is the ability to analyze the behavior of asphalt at the molecular scale and to characterize the relationship between molecular composition and macroscopic properties, such as oxidative aging [18,19,20], rejuvenation [21,22,23], interfacial adhesion [24,25,26,27], and molecular interaction [28,29]. MD has been applied to study the effects of WCO on the rejuvenation of aged asphalt. Yan [30] studied the effect of WCO (TG) on the adhesion properties of aged asphalt and aggregate, and found that WCO improved the resistance of aged asphalt to water damage. Wang [31] demonstrated that WCO (palmitic acid) promotes the diffusion of virgin and aged bitumen by reducing the viscosity of aged bitumen. Yu [32] studied the rejuvenation mechanism of WCO (FA) in aged SBS asphalt. Yan [33] evaluated the rejuvenation effects of WCO (TG) on aged asphalt in terms of the thermodynamic properties, molecular structure, diffusion behavior, adhesion properties, and water sensitivity of the asphalt binder–aggregate interface. Yan [34] investigated the influence of different stages of WCO on the thermodynamic properties of aged asphalt and found that treated fatty acids (TFA) were superior to fatty acids (FA).

Although there have been some studies on the effect of WCO on the rejuvenation of aged asphalt, their conclusions are not consistent due to the different molecular compositions (TG, FA) of WCO. A WCO with a high acid value has more recycling value due to its long-term usability. To date, for WCO with a fatty acid composition and a high acid value used over a long period of time, the evaluation of their rejuvenation effect on aged asphalt has been limited, and their rejuvenation mechanism remains unclear [32].

The objective of this study was to evaluate the effect of WCO with a high acid value on the properties of aged asphalt using molecular simulation. To comprehensively analyze the rejuvenation mechanism and effectiveness of the WCO rejuvenator on aged asphalt, the thermodynamic properties, molecular structure, adhesion work, and moisture susceptibility of the asphalt binder–aggregate interface were considered. The molecular models used included virgin asphalt, aged asphalt, and rejuvenated asphalt with different WCO contents, which were constructed based on the saturate, aromatic, resin, and asphaltene (SARA) four fractions. Density, fractional free volume, surface free energy, cohesion energy density, and solubility parameters were used to validate the models of different types of asphalt and to analyze their thermodynamic properties. The change in the asphalt’s colloidal structure and the rejuvenation mechanism were analyzed using the radial distribution function (RDF). The adhesion and debonding properties of the asphalt–mineral interface were evaluated using adhesion and debonding work, and the water sensitivity was quantified through the energy ratio.

## 2. Results and Discussion

To verify the effectiveness of the molecular simulation method and evaluate the rejuvenation effect of the WCO on the aged asphalt at the molecular level, the thermodynamic properties of the virgin asphalt, aged asphalt, and rejuvenated asphalt, including their density, cohesive energy density (CED), and free volume fraction (FFV), were calculated.

### 2.1. Density

**Figure 1:**
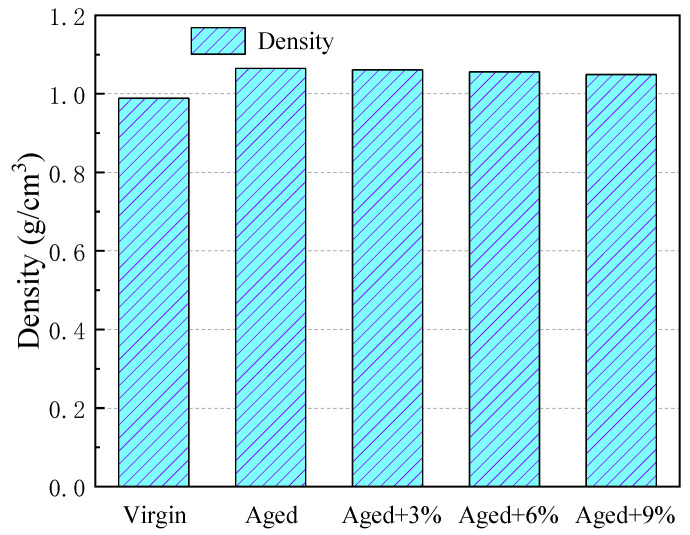
Densities of the different asphalt models at 298.15 K.

Density is an important thermodynamic parameter of asphalt and can be used as a direct indicator of the accuracy of simulation results. Figure 1 shows the density values for the virgin asphalt, aged asphalt, and rejuvenated asphalt with different WCO contents. As shown, the density of the virgin asphalt was about 0.99 g/cm^3^ and the density of aged asphalt was 1.07 g/cm^3^, which is in good agreement with Xu’s [20] and Gao’s [24] results and also agrees with the experimental results of 0.95–1.08 at 298.15 K found previously [35]. These simulation values are in good agreement with the available experimental values, which indicates that the established asphalt molecular model and calculation parameters are reasonable and can satisfy the required simulation accuracy well.

It can also be seen from Figure 1 that oxidative aging increases the density of the virgin asphalt due to the introduction of oxygen and sulfur atoms. The density of aged asphalt decreased with increases in WCO, and it was still higher than that of primary asphalt when 9% was added.

### 2.2. Cohesive Energy Density and Solubility Parameter

The cohesive energy density (CED) can be used to evaluate the intermolecular force within the asphalt model and reflect the interactions between functional groups. The higher the polarity of the groups in the molecule, the stronger the intermolecular force and the higher the CED. The solubility parameter SP can be used to evaluate the compatibility of the material and is the square of the CED. The equations for ED and SP are shown in Equations (1) and (2):

(1) $$CED=\frac{E_{coh}}{V}=\frac{-\langle E_{\mathrm{inter}}\rangle }{V}=\frac{E_{\mathrm{intra}}-E_{total}}{V}$$

(2) $$\mathrm{SP}=\sqrt{CED}$$

where *E_coh_* is the cohesive energy; V is the volume of a system; *E_inter_* is the intermolecular energy between all molecules; *E_intra_* is the intramolecular energy; and *E_total_* is the total energy of a system. The brackets represent the time average.

**Figure 2:**
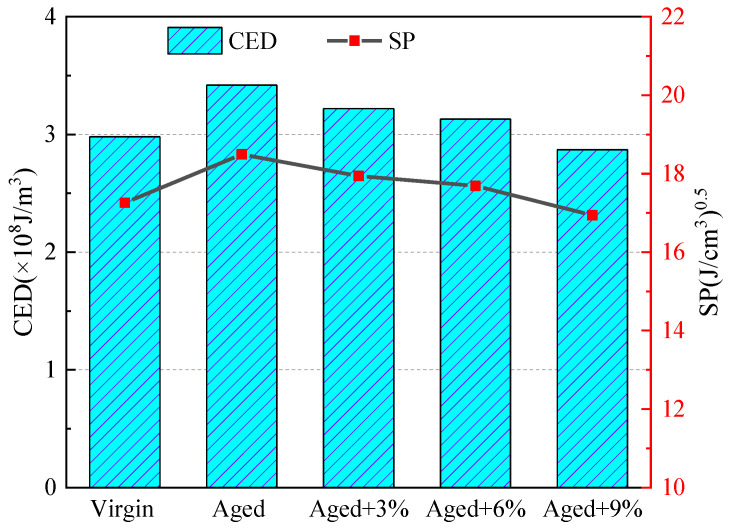
Cohesive energy density (CED) and SP values of the different asphalt models.

As shown in Figure 2, the CED and SP values of the virgin asphalt were in good agreement with the previously reported literature data [24,36]. It can be seen that with the aging of asphalt, CED and SP increased significantly, indicating that the intermolecular energy increased, resulting in the deterioration of internal molecular compatibility and diffusivity; this may be due to the introduction of ketones and sulfoxides increasing the molecular weight and polarity of the oxidized asphalt. In most cases, the greater the CED, the slower the molecular diffusion; thus, it is difficult for external molecules to penetrate the aged asphalt, which is manifested in the increase in asphalt viscosity on a macro level [37].

However, WCO can decrease the CED and SP values of the aged asphalt; that is, WCO can restore the compatibility properties of aged asphalt.

### 2.3. Fractional Free Volume (FFV)

According to the free volume theory, the volume of asphalt consists of free volume and occupied volume. The “occupied volume” represents a fixed volume that cannot be invaded by other molecules. However, in the free volume, the molecules can be rotated, twisted, and even flipped, ensuring that the asphalt has a certain fluidity. With increases in the free volume, the movement space of the molecules increases, thus improving the fluidity of the asphalt. In this study, the Connolly’s molecular surface area method [34,37,38] was used for the quantitative measurement of FFV, and the FFV calculation formula is given by Equation (3):

(3) $$\mathrm{FFV}=\frac{V_{free}}{V_{total}}=\frac{V_{free}}{V_{free}+V_{occupied}}\times 100\%$$

where *V_total_* is the total volume of the molecular model and *V_free_* and *V_occupied_* are the free volume and occupied volume, respectively.

**Figure 3:**
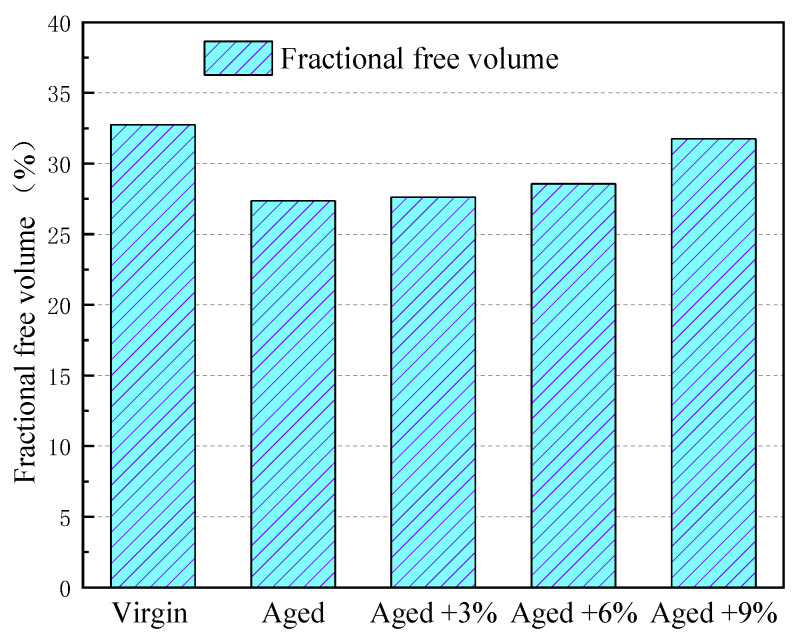
Fractional free volume (FFV) of different asphalt models.

The FFV is of great significance for the evaluation of the rheological properties, diffusivity, and glassy state behavior of asphalt [37]. Figure 3 compares the FFV of virgin, aged asphalt, and rejuvenated asphalt. The FFV of the asphalt decreased from 32.75% to 27.36% after aging, indicating that the asphalt structure became denser and reduced the free movement space of molecules. Therefore, a key reason for asphalt aging is the reduction in its free volume, which limits the thermal movement of its molecules, and the macro manifestation of this is that it becomes sticky and hard [39].

It can be seen from the figure that the FFV of aged asphalt increases with increases in WCO content due to the addition of lighter components. As shown in both Figure 2 and Figure 3, a higher FFV of asphalt coincides with a lower density and CED, which demonstrates weaker intermolecular interactions in the rejuvenated asphalt. It was therefore concluded that the addition of WCO can restore the rheological properties of aged asphalt.

### 2.4. Surface Free Energy

The theoretical definition of surface free energy (SFE) is the energy required to create a new surface per unit area in a vacuum, and it can be used to describe the degree of cohesion between molecules. In molecular dynamics, adding a vacuum layer to the asphalt cell can create a new surface.

The confined asphalt binder layer model was used to analyze the surface free energy and the work of adhesion to aggregates. By adding a 50 Å vacuum layer in the z direction of the constrained asphalt binder model, the confined asphalt binder layer model was established.

In this study, the surface energy was the ratio of the potential energy difference between the confined asphalt binder layer and bulk asphalt binder to the surface area, as shown in Equation (4) [22]:

(4) $$\gamma_{a}=\frac{\left(E_{film}-E_{bulk}\right)}{2A}$$

where *γ_a_* is the surface free energy; *E_film_* and *E_bulk_* are the potential energy of the confined asphalt layer model and bulk asphalt model, respectively, and *A* is the new Connolly’s molecular surface area to be created.

**Figure 4:**
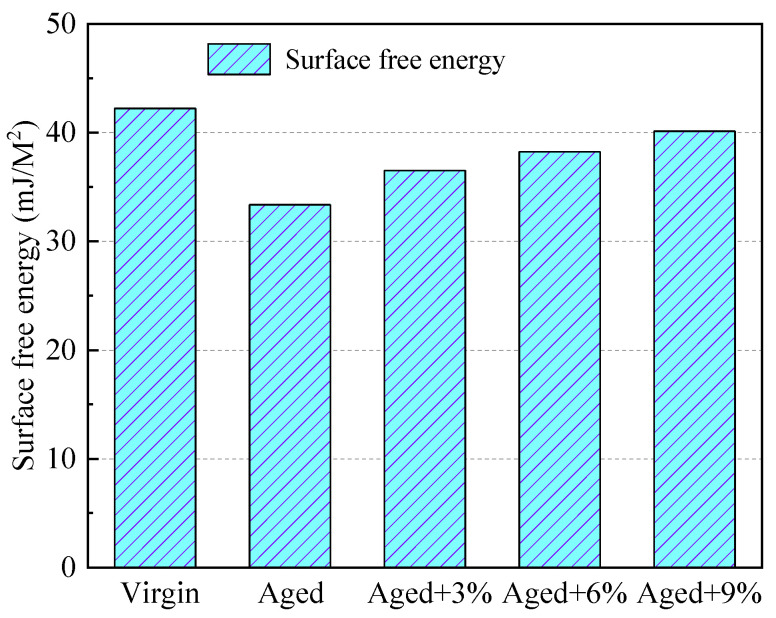
Surface free energy of different asphalt models.

The crack resistance of asphalt can be evaluated with the SFE; the higher the SFE, the more difficult the stripping of molecules and the better the crack resistance of asphalt will be. Figure 4 shows the results of the surface energy calculations for virgin, aged, and rejuvenated asphalt with different WCO contents. The calculated results for the virgin asphalt and aged asphalt agreed well with simulation values reported in the literature [20,22,35].

Figure 4 shows that the SFE of aged asphalt is significantly lower than that of virginal asphalt, indicating that there is weaker cohesion between molecules and that it is more prone to cracking. The main reason for this is that the asphaltene ratio of aged asphalt is relatively high, which reduces its surface free energy [40]. In addition, with increases in the WCO content, the SFE of aged asphalt increased, indicating that adding WCO to aged asphalt could enhance the cracking resistance of RAP. The main reason for this is that the addition of light components can restore the aged asphalt from a gel type to the sol–gel state of the original asphalt.

### 2.5. Radial Distribution Function

The microstructure of asphalt can be determined using the relative positions of its different components. The radial distribution function (RDF) describes the probability of finding another particle at a radial distance r from the target particle, similar to the ratio of the local density of the given molecule to the bulk density of the system. Therefore, the analysis of the asphalt microstructure using the RDF on the atomic scale provides a promising way of understanding the colloidal structure and rejuvenation mechanism of asphalt.

The radial distribution function is expressed as shown in Equation (5):

(5) $$g(r)=\frac{dN}{\rho 4\pi r^{2}dr}$$

In this study, the distribution of the SARA components in different asphalt models was analyzed using the RDF curve, where the distance is calculated based on the molecular centroid. The abscissa coordinate is the distance between molecules within the cutoff 25 Å, and the ordinate coordinate was *g*(*r*).

**Figure 5:**
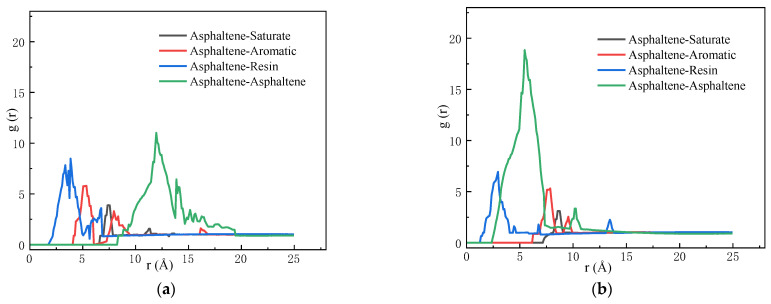
(**a**) Radial distribution functions for asphaltene with resin, aromatic, and saturate pairs in virgin asphalt. (**b**) Radial distribution functions for asphaltene with resin, aromatic, and saturate pairs in aged asphalt.

As shown in Figure 5a, the RDF of the asphaltene–asphaltene pairs showed the highest peak (around 12 Å) compared with the other SARA components, indicating strong self-aggregation behavior. The strong intermolecular aggregation of asphaltene is caused by the strong electrostatic interaction of oxygen atoms (asphaltene-phenol) and nitrogen atoms (asphaltene-pyro) in asphaltene [41,42]. Asphaltenes aggregate and form clusters or micelles over a wide range of concentrations and temperatures, which are the basis for the formation of asphalt colloidal structures [23]. The asphaltene–resin curve shows the closest peak around 3 Å, and the peaks of the asphaltene–saturate and asphaltene–aromatic curves are between the peaks of the asphaltene–asphaltene and asphaltene–resin curves. Therefore, the RDF of the SARA components indicates that the colloidal structure of asphalt is the resin adsorbed onto the surface of the asphaltene and dispersed in the maltene, consisting of saturate and aromatic components. This so-called asphalt sol–gel structure has been confirmed with experimental and simulated results [39,43,44].

The RDF curve of the SARA components in the aged asphalt is shown in Figure 5b. As seen when comparing Figure 5a,b, the peak value of the asphaltene–asphaltene curve increased significantly and the first peak position moved to the left, indicating that the self-aggregation of the aged asphalt was much more serious than that of the virgin asphalt. This may be due to the formation of higher-polarity carbonyl and sulfoxide functional groups in the aged asphalt, which intensified the aggregation between asphaltene molecules. For the asphaltene–resin curve, the peak coordinates of the asphaltene–resin shifted from the initial position (3.87, 3.19361) to a lower position of 4.41, 2.36877 after oxidative aging. This may be due to the high polarity of the aged asphaltenes, which reduced compatibility between the components and destroyed the colloidal structure of the asphalt. The intensification of asphaltene aggregation is the main reason for the degradation of asphalt properties [45,46].

**Figure 6:**
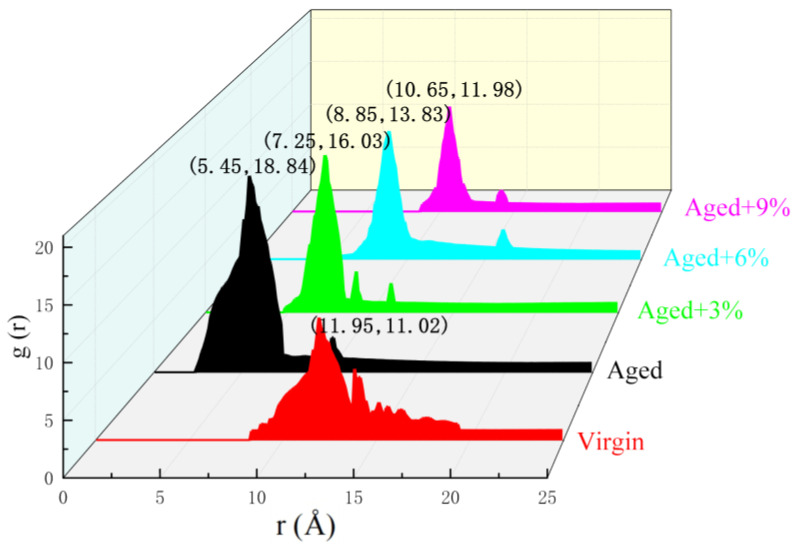
RDF curves of asphaltene–asphaltene pairs for the different asphalt models.

Therefore, it is necessary to analyze the aggregation behavior of asphaltene in the process of asphalt rejuvenation. To further study the effect of WCO content on asphaltene self-aggregation in rejuvenated asphalt, the asphaltene–asphaltene RDF curves for virgin asphalt, aged asphalt, and rejuvenated asphalt are shown in Figure 6.

The RDF curve shows a significant increase in the distance between asphaltene molecules and a decrease in the peak value after the addition of WCO. Therefore, the molecular self-aggregation between asphaltenes decreased, indicating that the distribution of asphaltene molecules in rejuvenated asphalt was more uniform than that in aged asphalt. Studies [47,48] have demonstrated that using the de-agglomeration effect of rejuvenators is the optimum way of restoring the properties of aged asphalt, which represents the true regeneration of aged asphalt.

**Figure 7:**
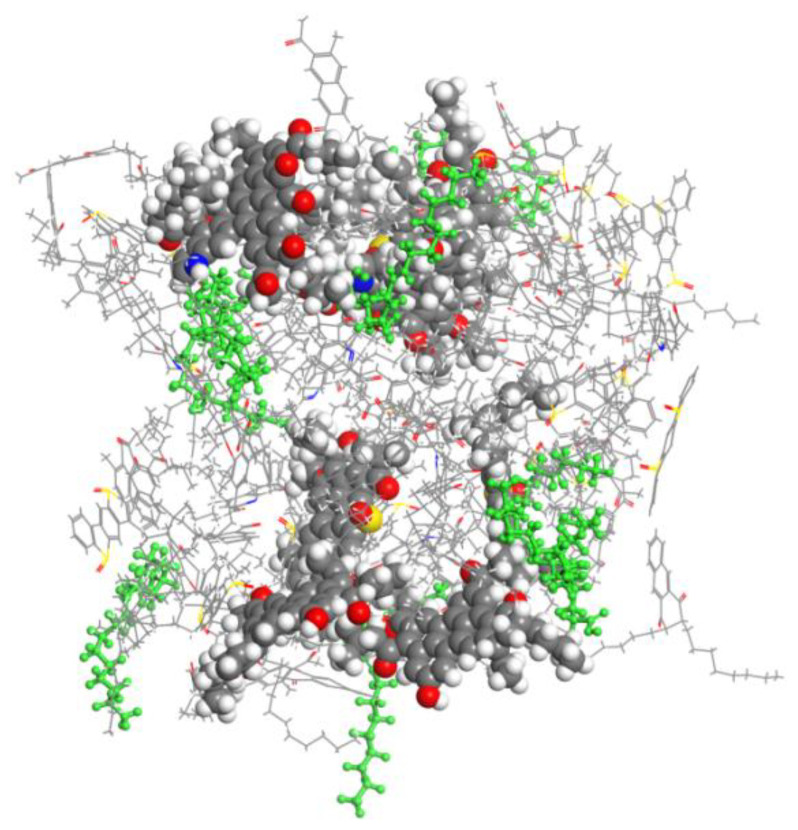
Spatial arrangement of WCO and asphaltene molecules. (CPK—asphaltene; green stick model—WCO molecule; linear model—other molecules).

Figure 7 shows the WCO molecular spatial arrangement of the rejuvenated asphalt steady-state structure. It can be seen that WCO molecules are distributed around the asphaltene, preventing its accumulation. The mechanism of the WCO rejuvenation of aged asphalt involves WCO molecules shielding the strong interactions between polarized groups, weakening the intermolecular forces of asphaltene and improving the colloidal structure of aged asphalt. Therefore, WCO does not reverse the oxidative aging process but restores the microstructure and improves the performance of the aging asphalt. This de-agglomeration of aged asphaltenes is a true regenerative process [17]. Therefore, WCO improves the performance of aged asphalt by restoring its microstructure, rather than by reversing the oxidative aging process.

### 2.6. Adhesion

The adhesion between asphalt and aggregate is the key factor in determining the visco-elastic and cracking properties of an asphalt mixture [49,50]. Therefore, the asphalt–aggregate interface requires strong and durable adhesion under complex traffic and environmental conditions.

Granite is commonly used as an aggregate in asphalt concrete and its main component is silica. In this study, silica was selected as an aggregate to study the adhesion properties of asphalt. The crystal structure of the silica was imported from the Materials Studio 2019 structure database. The SiO_2_ cell was first split in the [1,0,0] direction, and then the unit cell structure was repeated in the x and y directions. After this, a vacuum layer was added and a SiO_2_ mineral model was obtained, which was similar in size to the asphalt model. Considering that the silica surface had strong activity, a hydroxyl group with a density of 4.5 OH/nm^2^ was added to the silica surface to represent a fully hydrated silica substrate forming. 

The asphalt–SiO_2_ interface model was first geometrically optimized for 5000 iterations, followed by a dynamic equilibration run of 200 ps with the canonical ensemble (NVT) to ensure that the model configuration was further optimized. Then, the interaction energy and adhesion work were calculated using 50 ps NVT simulation.

In this study, adhesion work was used to quantify the adhesion between asphalt and aggregate interfaces; this is defined as the energy required to separate the asphalt–mineral interface per unit area into an asphalt surface and a mineral surface [25,51]. The work of adhesion (*W_adhesion_*) can be calculated with Equation (6) using the interaction energy (Δ*E_asphalt–aggregate_*), which is derived from Equation (7). A positive value of *W_adhesion_* indicates attraction between these two components, while a negative value of *W_adhesion_* indicates repulsion.

(6) $$W_{adhesion}=\frac{-\Delta E_{asphalt-aggregate}}{A}$$

(7) $$\Delta E_{asphalt-aggregate}=E_{total}-(E_{asphalt}+E_{aggregate})$$

where *W_adhesion_* is the work of adhesion between the asphalt and aggregate; Δ*E_asphalt–aggregate_* is the interaction energy between the asphalt and aggregate; *E_asphalt_* and *E_aggregate_* are the potential energy of an individual asphalt model and the aggregate surface model, respectively; *E_total_* is the total potential energy of the interface model; and *A* is the contact area between the mineral and the asphalt interface, which is calculated using the Connolly’s molecular surface area of the mineral surface.

**Figure 8:**
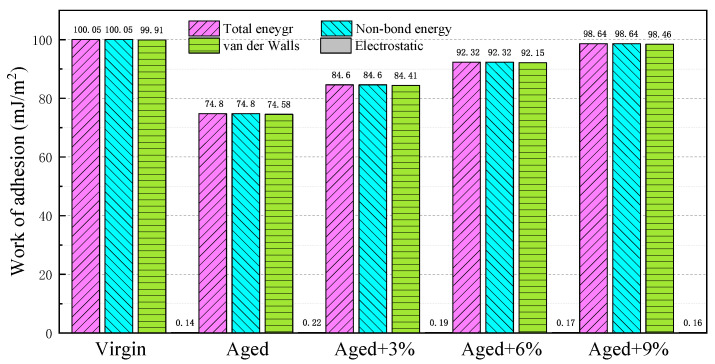
Composition of the adhesion work of the different asphalt–SiO_2_ interface models.

Figure 8 illustrates the energy components that contribute to the work of adhesion (*W_adhesion_*) between different asphalt models and SiO_2_. It was found that the total energy was equal to the non-bonding energy, which indicates that the adhesion between the asphalt and SiO_2_ is entirely caused by the non-bonding interaction, and that no chemical bond was formed. Meanwhile, the van der Waals interaction was the main component between the asphalt and SiO_2_ in the non-bonding energy components; this is because quartz is an electron-neutral mineral with very weak or no electrostatic interactions with other materials [25].

It can be seen from the figure that the adhesion work of the aging bitumen-SiO_2_ interface decreased. The fundamental reason for this is that the distance between the asphalt and quartz molecules increased, resulting in a significant decrease in van der Waals interaction [24]. With increases in the WCO content, the adhesion of asphalt to SiO_2_ increased, which mainly increased the van der Waals interaction. Together with Section 2.5, it was found that the positive effect of WCO on the adhesion between aged asphalt and SiO_2_ was mainly due to the restoration of the colloidal structure of the aged asphalt.

### 2.7. Work of Debonding

Due to the hydrophilic nature of the mineral and the hydrophobic nature of the asphalt, water intrudes into the asphalt mixture and weakens the bonds between the asphalt and the aggregate. It is necessary to study the water damage-resistance of WCO-rejuvenated asphalt on asphalt–aggregate interfaces via debonding experiments. 

The asphalt binder–water–aggregate interface model was constructed by adding 200 water molecules into the asphalt binder–aggregate interface model.

The work of debonding (*W_debonding_*), defined as the work required to displace bitumen with water on the asphalt–bitumen interface, is expressed by Equation (8) [25,51]:

(8) $$W_{debonding}=\frac{\left(E_{as-w}+\Delta E_{ag-w}-\Delta E_{as-ag}\right)}{A}$$

where *W_debonding_* is the work of debonding; Δ*E_as-w_* is the interaction energy between the asphalt and water; Δ*E_ag-w_* is the interaction energy between the aggregate and water; Δ*E_as-ag_* is the interaction energy between the asphalt and aggregate; and *A* is the interface contact area. A negative value of *W_debonding_* indicates that energy is released during the stripping process and that this process will occur spontaneously and without the need for external energy. Therefore, the higher the stripping work value, the more likely the asphalt is to be stripped from the mineral.

**Figure 9:**
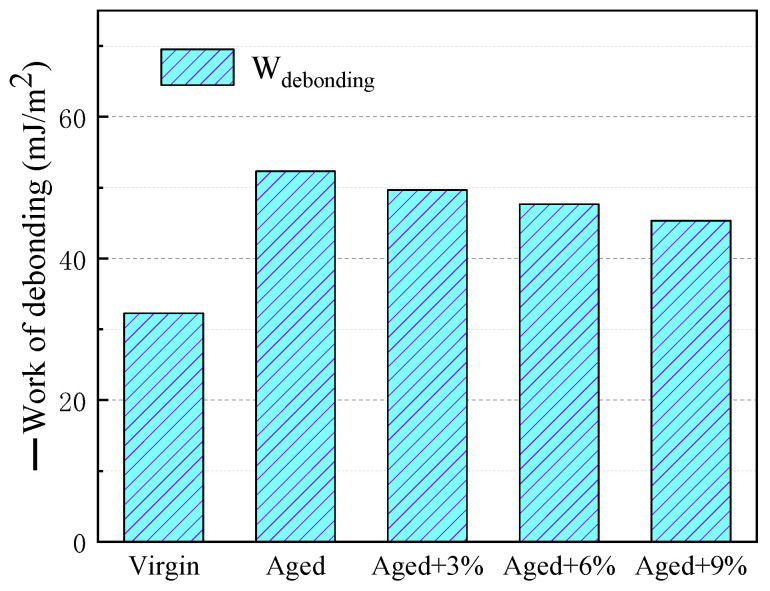
Work of debonding between different asphalt binders and SiO_2_.

The debonding work of different asphalt binders and SiO_2_ with water molecule interfaces can be seen in Figure 9. The work of debonding for all the asphalt–SiO_2_ systems was negative, which indicates that the stripping between asphalt and minerals occurs spontaneously and naturally. This calculation is consistent with laboratory tests showing that water intrusion occurs spontaneously in almost all asphalt–aggregate systems [25].

The debonding work of aged asphalt is higher than that of virgin asphalt, which indicates that it is easier to strip compared to virgin asphalt. With the addition of WCO, the debonding work of aged asphalt decreased to a certain extent, which indicates that the WCO increased the difficulty of stripping asphalt from SiO_2_.

### 2.8. Energy Ratio (ER)

The energy ratio is used to compare adhesion and debonding work and to quantify the water susceptibility of the asphalt binder–aggregate interface, as shown in Equation (9) in the MD simulation. The higher the *ER* value, the lower the sensitivity of the asphalt–aggregate interface system to water damage.

(9) $$ER=\left|W_{adhesion}/W_{debonding}\right|$$

where *W_adhesion_* is the work of adhesion between the asphalt and aggregate in dry conditions, and *W_debonding_* is the work of bonding when water displaces asphalt from the asphalt–aggregate interface.

**Figure 10:**
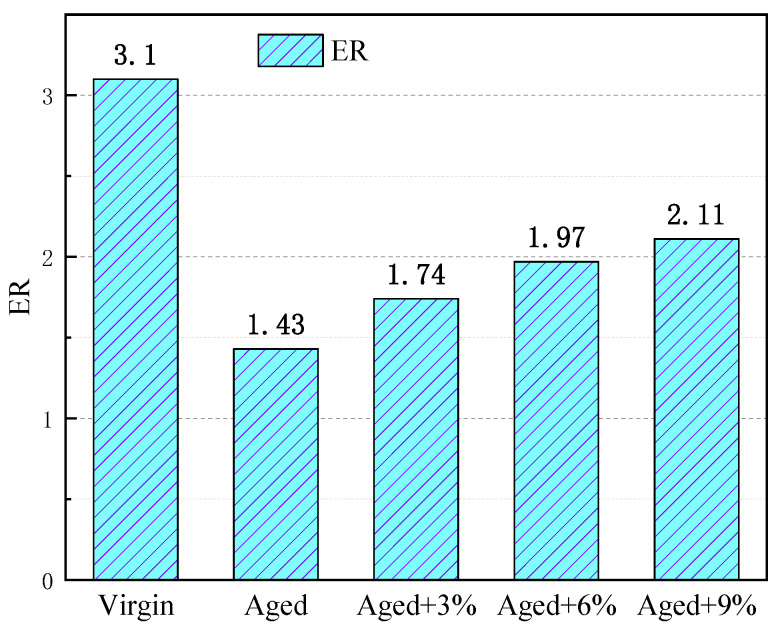
Energy ratio of the different asphalt binders and SiO_2_ interface models.

The ER values of the different asphalt–SiO_2_ interfaces are presented in Figure 10. It can be seen from the figure that the addition of WCO can reduce the water sensitivity of the interface between aged asphalt and SiO_2_, that is, WCO can improve the water damage resistance of the interface. However, the improvement effect of WCO on the water sensitivity of aging asphalt is limited. The simulation results are in agreement with the experimental results, which suggests that the water sensitivity of WCO-reclaimed asphalt should be improved [52,53]. The reason for this is the acidic nature of WCO, which limits or even weakens the water stability between the weakly acidic asphalt and neutral aggregate.

## 3. Materials and Methods

### 3.1. Molecular Structures of Asphalt Binders and Rejuvenators

**Figure 11:**
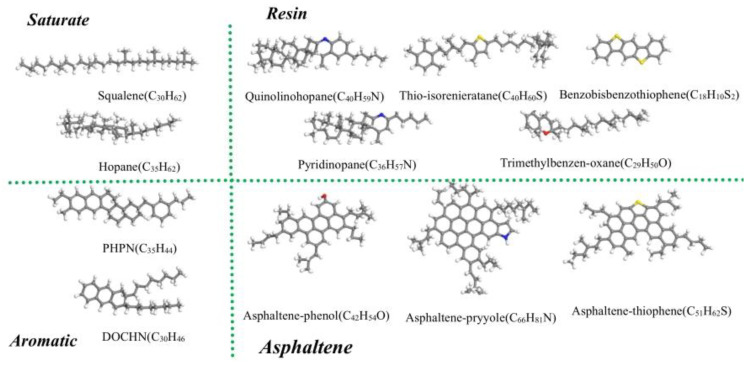
The 12-component molecular structure of virgin asphalt SARA fractions (Carbon atoms are grey, hydrogen atoms are white, sulfur atoms are yellow and red is oxygen).

To more accurately characterize the SARA components of asphalt molecules in terms of their elemental composition and Hansen solubility parameters, Li [54] improved the 12-component asphalt model of core asphalt AA-1 proposed by the Strategic Highway Research Program (SHRP). In the 12-component asphalt model, multiple-component molecules were proposed to represent each fraction of asphalt; it contained two types (squalane, hopane) for saturates (S), two types (Perhydrophe-nanthrene-naphthalene (PHPN), Dioctyl-cyclohexane-naphthalene (DOCHN)) for aromatics (A), five types (quinolinohopane, thio-isorenieratane, benzobisbenzothiophene, pyridinohopane, and trimethylbenzene-oxane) for resins (R), and three types (asphaltene-phenol, asphaltene-pyrrole, and asphaltenethiophene) for asphaltenes (A), as shown in Figure 11. The 12-component asphalt model has been adopted in many studies, and the rationality of the selection of these components has been well-proven [55,56]. Therefore, these chemical structures were selected in this study as the basis for building asphalt molecules.

It has been found that there are two types of readily oxidizable reactions in asphalt: one is the hydrogen atom attached to the phenyl carbon atom that is replaced by oxygen to produce a ketone, and the other is the reaction of sulfide with oxygen to produce sulfoxide [57,58]. Thus, ketones formed on phenyl carbon atoms and sulfoxides formed on sulfur atoms are used as representative aged asphalt molecules [19]. 

WCO used over a long period contains a large amount of free fatty acid (FFA) complexes, which is mainly related to the hydrolysis of TG in fresh oil during food frying. Oleic acid, linoleic acid, and palmitic acid (7:6:2) [59,60,61] are commonly used to analyze the microscopic interaction mechanisms between WCO with a high acid value and asphalt. Three WCO contents were investigated in the model by adjusting the number of molecules. The types and quantities of molecular models were compared and validated with the published literature.

### 3.2. Construction of Bulk Models

According to the molecular models of the virgin and aged asphalt components, bulk model systems of virgin asphalt and aged asphalt were constructed. The number and mass fraction of each component in the virgin asphalt and aged asphalt models are shown in Table 1. To analyze the effect of WCO on the microscopic properties of aged asphalt, a rejuvenated asphalt model with different WCO contents was established. The cell models of virgin, aged, and rejuvenated asphalt were constructed using an amorphous cell module in Materials Studio 2019. All the molecules were put into a large cubic cell according to their quantitative relationships to form a bulk bitumen model. The initial density of the cell was set as 0.1 g/cm^3^ to ensure the random distribution of the molecules. The system first underwent 50,000 steps of geometric optimization, followed by five annealing cycles of 100 ps between 298 K and 500 K to eliminate energy peaks. After this, the system performed dynamical calculations of 100 ps in the canonical ensemble (NVT) to reach equilibrium at the target temperature. Finally, the model was subjected to 100 ps kinetic calculations in an isotherm–isobaric (NPT) ensemble at 298 K and 0.1 MPa for relaxation and compression. The relaxation time of the NVT and NPT processes proved to be sufficient because the density, energy, and temperature of the model reached a stable state. The effectiveness of the simulation process was verified by comparing the calculated thermodynamic parameters with the literature and the experimental results.

### 3.3. Simulation Details

In this study, all the constructions and simulations of the model were performed using Materials Studio 2019 software. The Condensed-phase Optimized Molecular Potentials for Atomistic Simulation Studies (COMPASS) force field was the first ab initio force field that could effectively consolidate the atomic parameters of organic and inorganic materials. COMPASS II is an important development of the COMPASS force field in terms of atomic types and force field terminology, enabling the accurate prediction of the material properties of a wide range of separated and condensed compounds. It has been successfully applied for simulating asphalt binder in other studies [20,62,63].

During relaxation, the nose Hoover thermostat and Andersen barostat (MS 2019) were used in the model to maintain the target temperature and pressure, respectively. The summation method of the van der Waals interactions was atom-based, with a 15.5 Å cutoff distance. For electrostatic interactions, the Ewald summation method with a cutoff distance of 6 Å was used. 

## 4. Conclusions

In this study, the rejuvenation effect of WCO with a high acid value on aged asphalt was evaluated using MD simulation. Molecular models of virgin asphalt, aged asphalt, and rejuvenated asphalt with different WCO contents were developed. The thermodynamic properties, molecular structure, adhesion work, and moisture susceptibility of asphalt–SiO_2_ were analyzed. The main conclusions are listed as follows:

(1) WCO can improve the compatibility, rheology, and crack resistance of aged asphalt, and when the added amount is 9%, it is close to the level of primary asphalt.
(2) The mechanism of the WCO rejuvenation of aged asphalt was molecular de-aggregation. WCO molecules were distributed around the asphaltene, shielding the strong interactions between polarized groups, thus reducing the intermolecular forces and restoring the colloidal microstructure of the aged asphalt.
(3) WCO improves the interfacial adhesion between aged asphalt and SiO_2_, which was mainly manifested in the enhancement of the van der Waals interactions. WCO had a positive effect on the water damage resistance of the interface between aged asphalt and SiO_2_, although the effect was limited.

Future studies on the diffusivity of WCO molecules and their adhesion to other types of minerals are needed.

## Figures and Tables

**Table 1:** Molecular composition of virgin asphalt and aged asphalt.

SARA	Model Numbers	Virgin Asphalt		Aged Asphalt	
		Molecular Expression	Mass Fraction (%)	Molecular Expression	Mass Fraction (%)
Saturate	4	C_30_H_62_	11.11	C_30_H_62_	10.32
	4	C_35_H_62_		C_35_H_62_	
Aromatic	11	C_35_H_44_	31.90	C_35_H_36_O_4_	32.41
	13	C_30_H_46_		C_30_H_42_O_2_	
Resin	4	C_40_H_59_N	39.74	C_40_H_55_O_2_N	39.60
	4	C_40_H_60_S		C_40_H_56_O_3_S	
	15	C_18_H_10_S_2_		C_18_H_10_O_2_S_2_	
	4	C_36_H_57_N		C_36_H_53_O_2_N	
	5	C_29_H_50_O		C_29_H_48_O_2_	
Asphaltene	3	C_42_H_54_O	17.25	C_42_H_46_O_5_	17.67
	2	C_66_H_81_N		C_66_H_67_O_7_N	
	3	C_51_H_62_S		C_51_H_54_O_5_S	

## Data Availability

Data are contained within the article.
